# Transgenerational Effects of *p*CO_2_-Driven Ocean Acidification on Adult Mussels *Mytilus chilensis* Modulate Physiological Response to Multiple Stressors in Larvae

**DOI:** 10.3389/fphys.2018.01349

**Published:** 2018-10-15

**Authors:** Rosario Diaz, Marco A. Lardies, Fabián J. Tapia, Eduardo Tarifeño, Cristian A. Vargas

**Affiliations:** ^1^Graduate Program in Oceanography, Department of Oceanography, Universidad de Concepción, Concepción, Chile; ^2^Aquatic Ecosystem Functioning Laboratory (LAFE), Environmental Sciences Center EULA, Universidad de Concepción, Concepción, Chile; ^3^Department of Aquatic System, Faculty of Environmental Sciences, Universidad de Concepción, Concepción, Chile; ^4^Center for the Study of Multiple-Drivers on Marine Socio-Ecological Systems (MUSELS), Universidad de Concepción, Concepción, Chile; ^5^Millennium Institute of Oceanography, Universidad de Concepción, Concepción, Chile; ^6^Facultad de Artes Liberales, Universidad Adolfo Ibáñez, Santiago, Chile; ^7^Centro de Investigación Oceanográfica COPAS Sur-Austral, Universidad de Concepción, Concepción, Chile; ^8^Department of Zoology, Facultad de Ciencias Naturales y Oceanográficas, Universidad de Concepción, Concepción, Chile

**Keywords:** ocean acidification, transgenerational plasticity, multiple stressors, *Mytilus chilensis*, thermal effects

## Abstract

The effect of CO_2_-driven ocean acidification (OA) on marine biota has been extensively studied mostly on a single stage of the life cycle. However, the cumulative and population-level response to this global stressor may be biased due to transgenerational effects and their impacts on physiological plasticity. In this study, we exposed adult mussels *Mytilus chilensis* undergoing gametogenesis to two *p*CO_2_ levels (550 and 1200 μatm) for 16 weeks, aiming to understand if prolonged exposure of reproductive individuals to OA can affect the performance of their offspring, which, in turn, were reared under multiple stressors (*p*CO_2_, temperature, and dissolved cadmium). Our results indicate dependence between the level of *p*CO_2_ of the broodstock (i.e., parental effect) and the performance of larval stages in terms of growth and physiological rates, as a single effect of temperature. While main effects of *p*CO_2_ and cadmium were observed for larval growth and ingestion rates, respectively, the combined exposure to stressors had antagonistic effects. Moreover, we found a suppression of feeding activity in post-spawning broodstock upon high *p*CO_2_ conditions. Nevertheless, this observation was not reflected in the final weight of the broodstock and oocyte diameter. Due to the ecological and socioeconomic importance of mussels’ species around the globe, the potential implications of maternal effects for the physiology, survival, and recruitment of larvae under combined global-change stressors warrant further investigation.

## Introduction

The ocean is a natural sink for carbon dioxide (CO_2_); however, the increase of anthropogenic emissions is changing the ocean chemistry by lowering the seawater pH, and causing a reduction in the availability of carbonates (CO_3_^2-^) and biogenic calcium carbonate (CaCO_3_), a process widely known as ocean acidification (OA; Caldeira and Wickett, 2003; Feely et al., 2009; Gattuso and Hansson, 2011).

OA has been reported as a potential problem for calcifying organisms, limiting the production of biogenic calcium carbonate forms (Omega-Ω aragonite and calcite) and promoting their dissolution (e.g., Langdon et al., 2000; Caldeira and Wickett, 2003; Langdon and Atkinson, 2005; Kleypas et al., 2006; Hiebenthal et al., 2013). In the last decades, most of the research efforts studying OA have focused on its effects on marine biota (e.g., Gattuso and Hansson, 2011; Parker et al., 2013), reporting that early stages (embryos and larvae) of mollusks would be more sensitive to this global stressor (e.g., Kurihara, 2008; Gazeau et al., 2010; Waldbusser et al., 2014). However, most studies have focused on a single life stage, suggesting an acute exposure response at high *p*CO_2_ levels, without considering the effect of OA as a chronic process that can impact the biology of species throughout its life history (Welch and Munday, 2017) and modify the phenotypic traits of the offspring. This inheritance process is known as transgenerational plasticity and on occasion can constitute an adaptive mechanism transferred from parents to offspring exposed to particularly rigorous or stressful conditions, especially during gametogenesis process (Hamdoun and Epel, 2007; Byrne, 2011; Munday, 2014).

Different studies have been carried out on the effect of high *p*CO_2_ on the offspring of mollusks, suggesting a positive carryover of *p*CO_2_. For instance, a recent study where adults of the clam *Ruditapes philippinarum* were exposed to high *p*CO_2_ levels demonstrated an improvement in the offspring’s growth performance (Zhao et al., 2017). Similarly, larvae of the oyster *Saccostrea glomerata* grew to larger sizes and at a faster rate than those from lines reared under low CO_2_-control conditions (Parker et al., 2012). Similarly, adult oysters and their larvae have shown the same performance and higher capacity to modulate extracellular pH upon OA scenarios (Parker et al., 2015). The mussel *Mytilus edulis* not only increased the calcification performance in larvae stages reared at high *p*CO_2_ levels (Thomsen et al., 2017), but also changed their shell ultrastructure as an adaptive response, by precipitating only calcite, the more resistant form of CO_3_^2-^ (Fitzer et al., 2014). Nevertheless, transgenerational effects are not always positive. For instance, Hettinger et al. (2012) showed in Olympia oyster that OA was transmitted strongly across life stages and determined a deleterious impact manifested once larvae metamorphose and settle.

In a changing-ocean scenario, the interaction among multiple biotic and abiotic factors (Harley et al., 2006), and the organisms’ response to a combination of stress factors could be additive, antagonistic, or synergistic (Crain et al., 2008; Todgham and Stillman, 2013). The evidence of transgenerational plasticity in physiological performance in mollusks exposed to multiple stressors under OA scenarios is relatively scarce. Studies that exposed adults to additional factors in OA regimes indicate more sensitive larvae in the clam *Mercenaria mercenaria*, the scallop *Argopecten irradians* (Griffith and Gobler, 2017), and *S. glomerata* (Parker et al., 2017a) exposed to food limitation (Griffith and Gobler, 2017; Parker et al., 2017a), harmful algae (Griffith and Gobler, 2017), and low-salinity stress (Parker et al., 2017b).

Farming of Chilean mussels (*Mytilus chilensis*) is one of the leading industries in mussel production worldwide (FAO, 2014). Nevertheless, a significant problem for the mussel farming industry is the cadmium concentrations that regularly exceed the European standard (Figueroa, 2008; Sernapesca, 2015), which can lead to a rejection of global exports (Codex Alimentarius Commission [CAC], 2009). In the global change context, OA and temperature can modulate the sensitivity to trace-metal toxicity (Sokolova and Lannig, 2008; Nikinmaa, 2013; Ivanina and Sokolova, 2015). A synergistic effect of enhanced cadmium toxicity has been reported from experiments combining temperature, acidification, and cadmium as stressors for the scallop *Adamussium colbecki* (Benedetti et al., 2016) and the mediterranean mussel *Mytilus galloprovincialis* (Nardi et al., 2017).

Considering that variations in larval development can substantially impact the recruitment of mollusks (Calabrese et al., 1977; Machado and Lopes-Lima, 2011), hence the renewal and persistence of natural banks, the objective of this study was to assess the transgenerational carryover effect of OA in *M. chilensis*. Adult mussels conditioned for 4 months under current and projected future *p*CO_2_ levels were exposed to the interaction of temperature and sub-lethal concentrations of dissolved cadmium as additional stressors in a full cross-factorial design. The physiological traits considered as response variables in our study were egg diameter, larval size, and ingestion rates (IRs) in both larvae and adult mussels. Since feeding processes during early life stages of marine invertebrates are likely to be more sensitive to OA than in adults (e.g., Findlay et al., 2008, 2010), the influence of OA on feeding may explain observed impacts on these physiological traits and on other energy dependent processes including calcification (Vargas et al., 2013), and therefore it was considered a highly relevant physiological traits in our study.

## Materials and Methods

### Animal Collection

Adult specimens of the Chilean mussel, *M. chilensis* (67 ± 3 mm in shell length), were obtained from culture ropes (5 m depth) at a mussel-farming center located in Vilupulli, Chiloé, southern Chile (42° 35’ 35”S; 73° 47’ 18”W) during November 2015. The mussels were transported under wet conditions to the experimental laboratory at the University of Concepción’s Marine Biology Research Station in Dichato (Chile) and acclimatized for 3 weeks in 30 cm × 40 cm tanks filled with filtered seawater (0.1 μm + UV, pH = 8.1 ± 0.01, temperature = 13 ± 0.01°C, and salinity = ∼31 psu) and constant aeration. Throughout this acclimation period, mussels were fed daily with a phytoplankton suspension at saturation level (∼40 × 10^6^ cell mL^-1^, Phytogold-S, Castillo et al., 2017). Seawater was carefully renewed every 2 days.

### Seawater pCO_2_ Manipulation and Carbonate System Monitoring

To obtain the two different levels of seawater *p*CO_2_ (550–1200 μatm), dry air with pure CO_2_ was blended into each target concentration using mass flow controllers (MFCs) for both air and CO_2_. For each experimental tank, temperature, pH, and salinity were monitored every day while total alkalinity (TA) was measured every 10 days (**Table 1**). Samples for pH were collected in 50 mL syringes, avoiding formation of bubbles during collection and handling of the sample, and immediately transferred to a 25 mL thermostated closed cell at 25.0 ± 0.1°C for standardization (DOE, 1992; Torres et al., 2013), using a Metrohm^®^ 713 pH-meter with a glass combined double junction Ag/AgCl electrode (Metrohm model 6.0258.600). Samples for TA were stored in 500 mL borosilicate bottles (Pyrex, Corning^®^) and poisoned with 50 μL of saturated HgCl_2_ solution and with ground-glass stoppers lightly coated with Apiezon L^®^ grease. TA was determined using the open-cell titration method (Dickson et al., 2007), by using an automatic Alkalinity Tritrator Model AS-ALK2 Apollo SciTech. The AS-ALK2 system was equipped with a combination pH electrode (8102BNUWP, Thermo Scientific, United States) and temperature probe for temperature control (Star ATC probe, Thermo Scientific, United States) connected to a pH-meter (Orion Star A211 pH meter, Thermo Scientific, United States). All samples were analyzed at 25°C (±0.1°C) with temperature regulation using a water-bath (Lab Companion CW-05G). The accuracy was controlled against a certified reference material (CRM, supplied by Andrew Dickson, Scripps Institution of Oceanography, San Diego, CA, United States) and the TA repeatability was 2–3 μmol kg^-1^ in average. Temperature and salinity were measured using an Oakton SALT 6 + handheld salinity meter with probe (Salt6+, Oakton^®^, accuracy: ±1% and ±0.5°C, respectively). Temperature and salinity data were used to calculate the rest of the carbonate system parameters (e.g., *p*CO_2_, CO_3_^2-^) and the saturation stage of omega aragonite (Ω_aragonite_) and calcite (Ω_calcite_). Analyses were performed using CO2SYS software for MS Excel (Pierrot et al., 2006) set with Mehrbach solubility constants (Mehrbach et al., 1973) refitted by Dickson and Millero (1987). The KHSO_4_ equilibrium constant determined by Dickson (1990) was used for all calculations.

**Table 1:** Seawater characteristics (mean ± SD) used to rear both adult and larval stages of *M. chilensis* during the experimental period.

CO2 system parameters	Experimental treatments (nominal levels of *p*CO2)					
	550 (Exp 1#)	1200 (Exp 1#)	550 (Exp 2#)	1200 (Exp 2#)	550 (Exp 2#)	1200 (Exp 2#)
pH at 25°C (pH units)	7.80 ± 0.03	7.50 ± 0.03	7.80 ± 0.05	7.50 ± 0.04	7.75 ± 0.05	7.44 ± 0.03
pH *in situ* (pH units)	7.95 ± 0.03	7.63 ± 0.03	7.94 ± 0.05	7.60 ± 0.04	7.95 ± 0.06	7.62 ± 0.04
Temperature (°C)	15.00	15.00	15.00	15.00	11.00	11.00
Salinity (psu)	29.00	29.00	30.00	30.00	30.00	30.00
TA (μmol kg^-1^)	2135.60	2156.51	2270.90	2252.50	2270.90	2252.50
pCO2 *in situ* (μatm)	493.81 ± 35.98	1114.84 ± 85.25	528.57 ± 69.53	1134.14 ± 108.02	514.07 ± 75.91	1151.55 ± 108.00
[CO3^2-^] *in situ* (μatm kg^-1^)	109.09 ± 6.37	56.44 ± 3.83	118.13 ± 10.9	61.57 ± 5.15	104.76 ± 11.25	51.71 ± 4.47
Ωcalcite	2.70 ± 0.16	1.71 ± 0.10	2.91 ± 0.27	1.51 ± 0.13	2.57 ± 0.28	1.27 ± 0.11
Ωaragonite	1.40 ± 0.09	0.88 ± 0.06	1.83 ± 0.17	0.96 ± 0.08	1.62 ± 0.17	0.80 ± 0.07

### Preliminary Toxicity Experiment (Experiment 1#)

**FIGURE 1:**
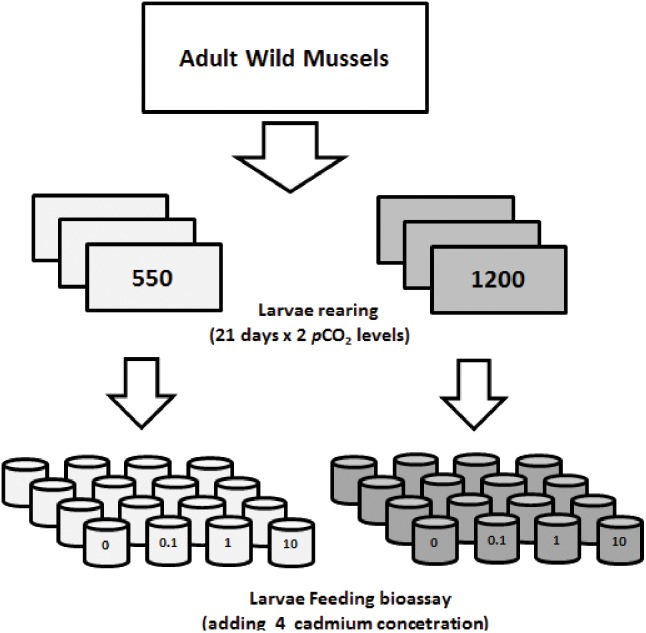
Schematic of Experiment #1, used to determine the feeding response of 21 days old larvae rearing under two *p*CO_2_ levels (gray: 550 μatm and dark: 1200 μatm), adding nominal concentrations of dissolved cadmium (Cd^+2^: 0–0.01–1 and 10 μg L^-1^) in a 24-h bioassay. Four replicates were considered for ingestion rate estimates (*n* = 4).

A preliminary experiment was carried out to determine the levels of cadmium selected for the transgenerational carryover and multiple-driver experiment. Broodstock were induced to spawn through heat shock technique (Bayne, 1976). Two females and two males were effectively spawned per experiment. The gametes were homogenized and arranged in densities of 10 oocytes L^-1^ in the different treatments and then, we have added a concentration of spermatozoids maintained at the same *p*CO_2_ condition of the corresponding broodstock. Larvae were obtained following Ruiz et al. (2008) and Toro et al. (2012). A larval culture was performed at initial density of 10 larvae mL^-1^ in 1 L acid-washed borosilicate bottles (Duran Schott^®^) during 21 days, and at two *p*CO_2_ levels: (i) present conditions in the mussel farming area (control: 550 μatm, *n* = 3) and (ii) the worst case scenarios (IPCC A2 emission scenario) predicted for 2100 (1200 μatm, *n* = 3; Meinshausen et al., 2011; **Figure 1**). Present *p*CO_2_ conditions were established based on a time-series analysis of data collected by an oceanographic buoy deployed by the Center for the Study of Multiple Drivers on Marine Socio-Ecological Systems (MUSELS) in the same mussel-farming site^1^ (Vargas et al., 2017). We have assumed the projections for 2100 in this coastal waters, as the additive effect of the difference expected for the open ocean in equilibrium with the projected atmospheric levels (from ∼400 to 1000 μatm = 600 μatm), which for coastal waters (550 μatm) corresponds to an average value between 1150 and 1200 μatm. During this larval rearing experiment, seawater was renewed every two days (0.1 μm + UV) using pre-equilibrated *p*CO_2_ water. Temperature was controlled by a thermoregulated bath (15°C) and larvae fed daily ∼3.0 μg chlorophyll a (Chl-a) L^-1^ (∼400.000 cells mL^-1^) of *Isochrysis galbana.*

After the 21 days rearing, a 24-h bioassay was performed to assess feeding rates of larvae (see section “Ingestion Rate Experiments”) and exposed to a nominal concentration of dissolved cadmium Cd^+2^: 0, 0.01, 1, and 10 μg L^-1^ added to each *p*CO_2_ level. Four replicates were considered for IR estimates. We have considered these ranges based on natural concentrations observed in coastal environments along the Chilean coast and a realistic polluted scenario (Neff, 2002).

### Transgenerational Carryover and Multiple Driver Experiment (Experiment 2#)

**FIGURE 2:**
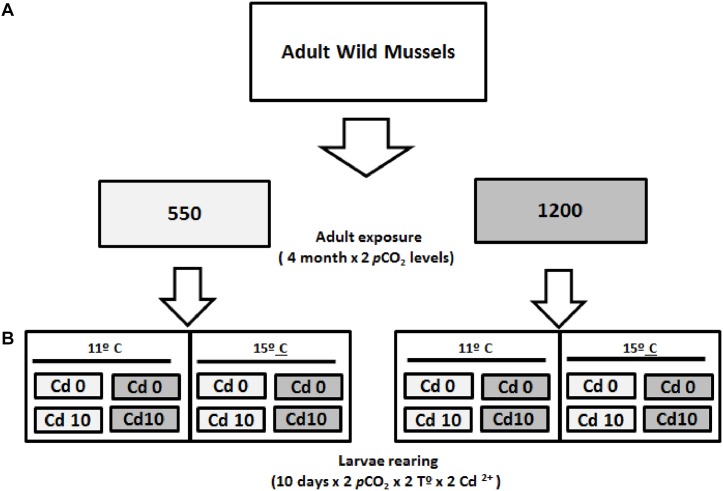
Schematic of Experiment #2, used to investigate the combined consequences of transgenerational effect and multiple stressors on *M. chilensis* larvae: *p*CO_2_ (gray: 550 μatm and dark: 1200 μatm), temperature (11–15°C), and cadmium (Cd0: 0 μg L^-1^ and Cd10: 10 μg L^-1^). **(A)** Adult acclimatization in two *p*CO_2_levels. **(B)** Schematic of larvae rearing (see section “Materials and Methods” for further details).

Spawned adult mussels were stabilized for 1 week under acclimation conditions (see above), and then moved into one of two *p*CO_2_ levels (550 and 1200 μatm). Mussels were kept in eight 25 L tanks with five specimens each (mean density = 0.2 ind L^-1^). Temperature was increased to 15°C to promote gonadal development (Lagos et al., 2012b). After 16 weeks of incubation at these nominal *p*CO_2_ levels, each group of adult mussels was induced to spawn maintaining the same low and high *p*CO_2_ conditions. Fertilization and larval stages from each group were reared at the *p*CO_2_ treatments, thus completing a full cross-factorial design (2 × 2 × 2); i.e., two *p*CO_2_ levels (550–1200 μatm), two nominal concentrations of dissolved Cd^+2^ (0–10 μg L^-1^), and two temperature levels representing the spring–summer environmental range at the mussel farming site (11–15°C; **Figure 2**).

Fertilization and larval rearing were carried out in 1 L borosilicate bottles (three replicates per group, initial density = 10 ind mL^-1^). Seawater was renewed every 2 days and larval stages were fed daily at ∼3.0 μg Chl-a L^-1^ (∼400.000 cells mL^-1^) of *I. galbana*.

Subsamples of hydrated oocytes were collected from each *p*CO_2_ treatment and preserved in a 3% phosphate buffered formalin solution for egg-diameter measurements using an inverted microscope. At days 4, 8, and 11, subsamples of current larvae rearing were also collected and fixed in 70% ethanol for measurements of shell length and larval growth estimates. At days 4 and 11, individuals were also collected for larval feeding experiments.

Finally, spawned broodstock were returned to the culture tanks and maintained at the same *p*CO_2_ conditioning levels for three weeks, in order to compare the total weight (g), the metabolically active tissue (g), and IR for both treatments after this recovery time post-spawning.

### Ingestion Rate Experiments

Ingestion rates were measured as Chl-a removal using a static system the experimental procedure outlined by Coughlan (1969). Clearance and ingestion-ratios calculations were conducted according to Frost (1975) and modified by Marin et al. (1986). For larval stages, 25 individuals were collected and transferred to 200 mL acid-washed polycarbonate bottles (0.13 ind mL^-1^) filled with the corresponding treatment of *p*CO_2_ levels, temperature, Cd^+2^ concentration, and subsequently fed with *I. galbana* (∼3.0 μg Chl-a L^-1^). Three control bottles without larvae and four bottles with larvae for each corresponding treatment were incubated for approximately 20 h and periodically rotated by hand to avoid particle sedimentation. Similar procedures were followed for adult mussels, which were individually incubated for 4 h in 2 L baskets with around 3 mL of dry food (∼7.0 μg Chl-a L^-^1).

Ingestion rate was estimated as the change in food concentration as indicated by Chl-a concentration. Upon incubation, 100 mL subsamples were filtered (0.7 μm) and extracted in acetone 95% at dark before the measurement on a TD 700 Turner fluorometer (Strickland and Parsons, 1968). For adult feeding estimates, total wet weight (TW) and buoyant weight (BW) were determined with an analytical balance (±0.01 mg) to estimate the metabolically active tissue (TW–BW; Lardies et al., 2017). IR in adult mussels was standardized per metabolically active tissue (μg Chl-a g^-1^ h^-1^) and for larvae (ng Chl-a larva^-1^ h^-1^). Negative IRs were not included in our statistical analyses, and we always used a minimum of two replicates.

### Statistical Analyses

Two-way ANOVA was used to evaluate larvae IR tests (preliminary toxicity experiment) and one-way ANOVA for adult TW, MT, and oocyte diameter (transgenerational experiment). Interaction between transgenerational carryover (adult *p*CO_2_ exposure) and multiple drivers (*p*CO_2_, temperature, Cd^+2^) on larval growth (shell length) were tested by factorial nested ANCOVA with larval age (i.e., culture time) as covariate and a factorial nested ANOVA for IRs adding larval age as a factor. Factors analyzed were nested in adult *p*CO_2_ exposure. When the analysis showed significant interactions, multiple comparisons were carried out using Tukey’s *a posteriori* HSD test on each factor that showed significant differences using a Bonferroni correction. Assumptions of normality and homoscedasticity for the ANOVA’s test were evaluated using the Kolmogorov–Smirnov and Bartlett tests, respectively (Zar, 2010), and square-root transformation was applied when necessary (**Supplementary Material**). All analyses were carried out using Statistica version 7.0 software.

## Results

### Preliminary Toxicity Experiment (Experiment 1#)

**FIGURE 3:**
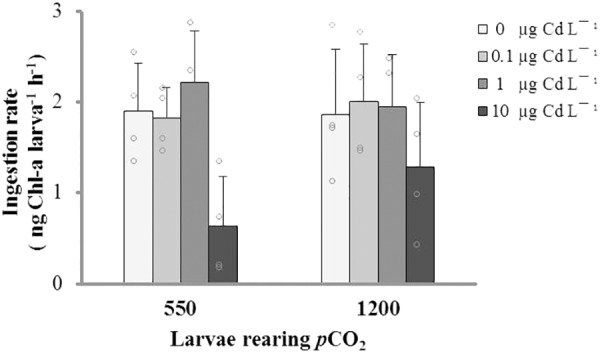
Ingestion rate (mean ± SD) of *M. chilensis* larvae reared for 21 days under two *p*CO_2_ levels (550 and 1200 μatm) and exposed to four nominal concentrations of dissolved cadmium in a 24-h bioassay.

The results of our acute physiological response experiment with 21-days-old larvae evidenced that IRs were not significantly different among *p*CO_2_ treatments, but the 24-h exposure to 10 μg L^-1^ Cd^+2^ significantly reduced the IR in larval stages from both *p*CO_2_ levels (**Figure 3** and **Table 2**).

**Table 2:** Results of two-way ANOVA for testing variation in ingestion rates in *M. chilensis* larvae reared for 21 days under two different *p*CO_2_ levels (550 and 1200 μatm) and exposed to nominal concentrations of dissolved cadmium (Cd^+2^: 0, 0.01, 1, and 10 μg L^-1^) in a 24-h bioassay.

Variable	Factor	F	*d.f./d.f_total_*	*p*-value	Tukey *post hoc* comparisons
Ingestion	*p*CO_2_	0.41	1,24	0.53	
	Cd^+2^	6.43	3,24	**<0.01**	10 ≠ all
	*p*CO_2_ × Cd^+2^	0.71	3,24	0.55	

*Bold values represent statistically significant p-value.*

### Transgenerational Carryover and Multiple Driver Experiment (Experiment 2#)

#### Physiological Response of Adult Mussels Conditioning Upon High pCO_2_

**FIGURE 4:**
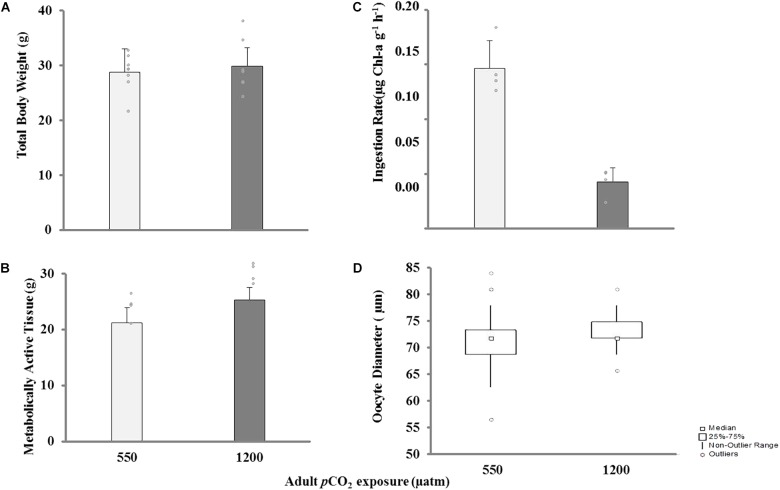
Biological response of adult mussels under two *p*CO_2_ conditions (550 and 1200 μatm): **(A)** total wet weight, **(B)** metabolically active tissue, **(C)** ingestion rate, and **(D)** oocyte diameter, after 4 months of experimental rearing in two *p*CO_2_ levels (550 and 1200 μatm; mean ± SD) and 3 weeks of post-spawning recovery (mean ± SD).

The statistical comparison of the biological response in adult mussels conditioned under high *p*CO_2_ is shown in **Table 3**. Adult mussels conditioned during 4 months at two *p*CO_2_ levels did not exhibit significant post-spawning differences in either total weight (**Figure 4A**) or metabolically active tissue (**Figure 4B**). However, mean IR in the control group (550 μatm) was significantly higher than under high *p*CO_2_ conditions (1200 μatm; **Figure 4C**), which in turn suggests a metabolic depression and lower capacity for post-spawning recovery in adult mussels. In both cases, oocyte diameters were not significantly different from the *p*CO_2_ treatment exposure of broodstock (**Figure 4D**).

**Table 3:** Statistical results of one-way ANOVA of adult mussels at the end of the experiment: total wet weight (g), metabolically active tissue (g), ingestion rate, and oocyte diameter (μm) after 4 months exposure to two *p*CO_2_ levels (550 and 1200 μatm).

Variable	Factor	F	*d.f./d.f_total_*	*p*-value	Tukey *post hoc* comparisons
Total wet weight	*p*CO_2_	4.50	1,13	0.61	
Metabolically active tissue	*p*CO_2_	2.86	1,7	0.13	
Ingestion rate	*p*CO_2_	15.18	1,6	**<0.01**	550 ≠ 1200
Oocyte diameter	*p*CO_2_	1.43	1,126	0.23	

*Bold values represent statistically significant p-value.*

#### Physiological Response of M. chilensis Larvae Upon Transgenerational Effect and Multiple Stressors

**FIGURE 5:**
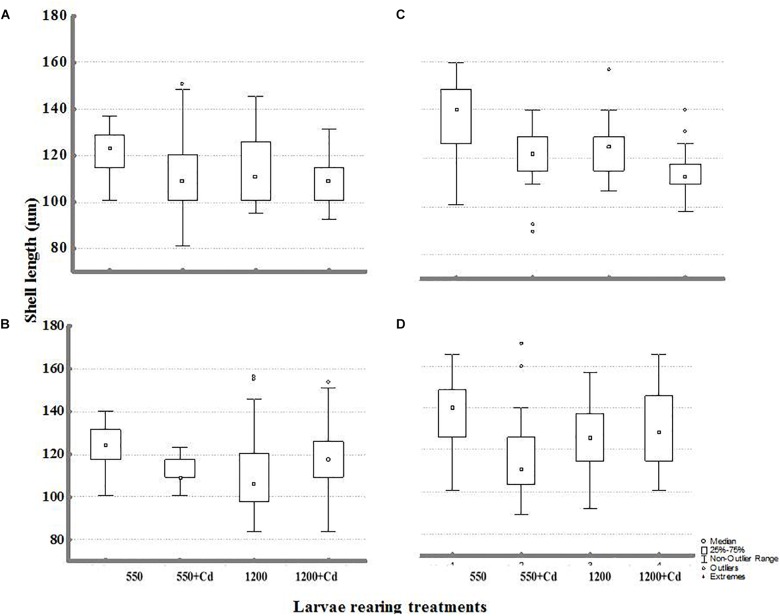
Shell length of larvae obtained from adults conditioned under two *p*CO_2_ levels (550 and 1200 μatm) after 11 days of rearing at multiple combinations of *p*CO_2_ (550 and 1200 μatm), temperature (11 and –15°C), and cadmium concentration (0 and 10 μg L^-1^): **(A)** larval rearing at 11°C and **(B)** larval rearing at 15°C from parents conditioned at 550 μatm; **(C)** larval rearing at 11 and **(D)** 15°C from parents conditioned at 1200 μatm.

Statistical comparisons of the effect of multiple stressors on larval growth (shell length) and IRs under experimental conditions are shown in **Tables 4**, **5**. Larval shell length (**Figures 5A–D**) was significantly different from broodstock conditioned to different *p*CO_2_ levels, with larger larvae resulting from broodstock exposed to high *p*CO_2_ condition, and larval stages cultured upon low *p*CO_2_ levels. Moreover, the parental effect conditioned the response of mussel larvae upon contrasting *p*CO_2_ and temperature conditions (**Table 4**). *Post hoc* analysis indicated evidence of larger shell sizes in offspring from broodstock exposed to high *p*CO_2_ levels, and larval rearing under high temperature and low *p*CO_2_ treatment.

**Table 4:** Results of nested ANCOVA: effect of adult *M. chilensis p*CO_2_ exposure (550 and 1200 μatm) on the shell length (μm) of larvae reared under multiple stressors: *p*CO_2_ (550 and 1200 μatm), temperature (11 and 15°C), and cadmium (0 and 10 μg L^-1^).

Variable	Factor	F	*d.f./d.f_total_*	*p*-value	Tukey *post hoc* comparisons
Shell length	Broodstock	29.45	1,1423	**<0.01**	550 ≠ 1200
	*p*CO_2_ (Broodstock)	4.43	1,1423	**0.04**	550 (1200) ≠ All
	T° (Broodstock)	4.88	1,1423	**0.02**	15° (1200) ≠ 11–15° (550) 15° (550) ≠ 11° (1200)
	Cd^+2^ (Broodstock)	2.66	1,1423	0.10	
	*p*CO_2_ × T°	2.17	1,1423	0.14	
	*p*CO_2_ × Cd ^+2^	16.78	1,1423	**<0.01**	0 (550) ≠ all
	T° × Cd^+2^	0.00	1,1423	0.94	

*Bold values represent statistically significant p-value.*

**Table 5:** Results of nested ANOVA: effect of adult *M. chilensis p*CO_2_ exposure (550 and 1200 μatm) on feeding response of larvae reared under multiple stressors: *p*CO_2_ (550 and 1200 μatm), temperature (11 and 15°C), and cadmium (0 and 10 μg L^-1^).

Variable	Factor	F	*d.f./d.f_total_*	*p*-value	Tukey *post hoc* comparisons
Ingestion Rate	Broodstock	6.42	1,60	**0.01**	550 ≠ 1200
	*p*CO_2_ (Broodstock)	0.002	1,60	0.96	
	Age (Broodstock)	1.20	1,60	0.28	
	T° (Broodstock)	7.07	1,60	**<0.01**	11 (1200) ≠ all
	Cd^+2^ (Broodstock)	4.06	1,60	**0.05**	0 (550) ≠ 0 (1200) **0 (550) ≠ 10 (1200**
	*p*CO_2_ × age	0.15	1,60	0.70	
	*p*CO_2_ × T°	0.32	1,60	0.58	
	*p*CO_2_ × Cd^+2^	3.42	1,60	0.07	
	Age × T°	10.03	1,60	**< 0.01**	4 (11°) ≠ 10 (11°) 10 (15°) ≠ all
	Age × Cd^+2^	0.088	1,60	0.77	
‘	T° × Cd^+2^	0.199	1,60	0.66	
	*p*CO_2_ × age × T°	2.74	1,60	0.10	
	*p*CO_2_ × age × Cd^+2^	0.069	1,60	0.794	
	*p*CO_2_ × T° × Cd^+2^	3.97	1,60	0.051	
	Age × T° × Cd^+2^	0.46	1,60	0.499	
	*p*CO_2_ × age × T° × Cd^+2^	0.68	1,60	0.41	

*Bold values represent statitiscally significant p-value.*

On the other hand, cadmium had a non-significant effect on larval size. A greater shell length in those larvae reared at low *p*CO_2_ and non-Cd^+2^ additions was evidenced from the factorial interactions. The interactions between *p*CO_2_ with temperature and Cd^+2^ were non-significant in shell length of progeny.

**FIGURE 6:**
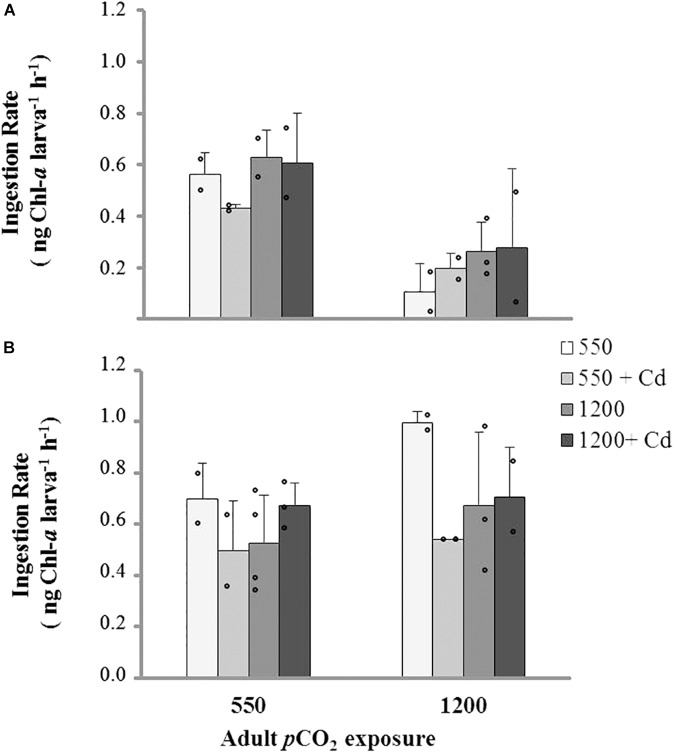
Ingestion rate (mean ± SD) of larvae obtained from adults conditioned under two *p*CO_2_ levels (550 and 1200 μatm) after 11 days of rearing at multiple combinations of *p*CO_2_ (550 and 1200 μatm), temperature (11 and 15°C), and cadmium concentration (0 and 10 μg L^-1^): (**A**) larval rearing at 11°C and (**B**) larval rearing at 15°C.

Ingestion rates in larvae were also significantly related to *p*CO_2_ exposure in broodstock, and their interaction with temperature and Cd^+2^. No differences were found in relation to larval rearing at changing *p*CO_2_ conditions as observed in the preliminary experiment on IRs (**Figure 6**). *Post hoc* tests showed lower IRs in the offspring of high-*p*CO_2_ adults when reared at 11°C (**Figure 6A**) and an increase of IRs in larvae from adults conditioned under control *p*CO_2_ levels and without Cd^+2^ exposure. Multistressor interactions only indicated significant differences between temperature and larvae culture age, showing a higher IR in 11-days-old larvae reared at 15°C (**Figure 6B**), the rest of the treatment combinations showed non-significant differences (**Figure 6**).

## Discussion

Our results indicate that combinations of different stressors found in the coastal ocean may interact antagonistically as far as the physiology of mussels is concerned, with the parental effect being a significant factor in the physiological performance of mussel larvae, and temperature being a key variable that affects both the growth and feeding of offspring. The specific global change stressor (OA), which is maintained in the broodstock evidently, has important consequences for adult energy budgets and the physiological performance of their offspring.

Our results demonstrated that oocyte size in both *p*CO_2_ levels were smaller (∼2 μm) than reported by Lagos et al. (2012b) for *M. chilensis* but within the range reported by Ockelmann (1965) for a number of bivalves. No effect in oocyte size from broodstock exposed to high *p*CO_2_ levels was observed. Similar results have been observed in transgenerational studies in the sea urchin *Strongylocentrotus droebachiensis* (Dupont et al., 2013), the clam *M. mercenaria*, the scallop *A. irradians* (Griffith and Gobler, 2017), and the oyster *S. glomerata* (Parker et al., 2017a).

We report the effect of OA on the post-spawning recovery of organisms due to their high-energy demand. The weight of the broodstock was similar in both *p*CO_2_ levels (wet weight and metabolically active tissue), but the feeding performance was substantially higher in specimens exposed to control *p*CO_2_ levels. Kurihara et al. (2013) found a delayed reproductive phenology in sea urchin *Hemicentrotus pulcherrimus* and reduced energy intake in futuristic *p*CO_2_ levels. In the copepod *Acartia steueri*, egg production rates decreased significantly under high *p*CO_2_ (Kurihara et al., 2004), whereas in the marine shrimp *Palaemon pacificus*, egg production was suppressed but feeding was not affected by *p*CO_2_ treatments (Kurihara et al., 2008). The effect of OA in the post-spawning recuperation of adult organisms could determine the success of the subsequent spawning period, and consequently affect the life-history cycle as well as population renewal. Furthermore, mussels exposed to high *p*CO_2_ for a long time show a plastic response because they are capable of physiologically compensating the drop in feeding rate, while maintaining growth and tissue stable under these stressful conditions. We hypothesize that mussels exposed to high *p*CO_2_ levels could have experienced a drop in their respiration rates (and/or excretion), reducing the energy expenditure via these processes. Indeed, Navarro et al. (2013) showed that *M. chilensis* reduced both the clearance/ingestion and respiration rates upon high *p*CO_2_ conditions. In consequence, mussels could be able to compensate the reduction in food intake by reducing their metabolic costs in other processes (growth rates) together with an increase in the absorption efficiency.

Our experimental approach considered an initial spawning of adult mussels in order to homogenize the state of gonadal development prior to reproductive conditioning under two OA scenarios (550–1200 *p*CO_2_) and otherwise optimal conditions of food supply and temperature. While previous experimental studies in *M. chilensis* have reported conditioning periods of up to 81 days at 15°C (Lagos et al., 2012b), we extended this period to ca. 4 months to be consistent with other transgenerational OA experiments in mollusks (Parker et al., 2012, 2017a; Fitzer et al., 2014; Griffith and Gobler, 2017; Zhao et al., 2017).

Parental effects were significant in the physiological response of larvae upon exposure to multiple drivers. Both shell length and IR in mussel larvae depend directly on the broodstock *p*CO_2_ exposure. In consequence, the potential effect of *p*CO_2_-driven OA could change through different generations of mussel populations (Duarte et al., 2014; Osores et al., 2017). Osores et al. (2017) reported that the phenotypic plasticity in the feeding rates was related to the environmental component, whereas the growth rate remains independent, which in turn suggests the importance of a genetic component. In our study, shell length was smaller than values reported by Ruiz et al. (2008), which suggests that it could have been attributed to differences in reared system and food supply (Lagos et al., 2012a).

Larval size is related to the fitness of individual offspring in many invertebrates (Bernardo, 1996; Fox and Czesak, 2000), including bivalves (Przeslawski and Webb, 2009; Ventura et al., 2016). In nature, a smaller initial size could result in higher juvenile mortality for at least two reasons. First, smaller young may have lower energy reserves and, and consequently would be more sensitive to periodic food shortages in the sea (see Phillips, 2002). Second, and probably a more important effect of smaller initial size, is that the juveniles remain small for a longer time. Consequently, these smaller juveniles are exposed to a greater predation risk (Rumrill, 1990; Johnson and Smee, 2012). That is, OA can significantly affect larval survival, and at the same time affect the broodstock fitness.

Our study also showed that larger larvae were commonly observed when reared at low *p*CO_2_ conditions. A similar effect has been found in other transgenerational studies in others mollusks (Parker et al., 2012; Griffith and Gobler, 2017). Greater larval performance under current *p*CO_2_ conditions may stem from greater ease to calcify due to greater availability of biogenic carbonates, which would agree with previous experimental studies (e.g., Kurihara et al., 2007; Kurihara et al., 2008; Gazeau et al., 2010); however, results are not directly comparable as these studies did not consider parental acclimation in acidification.

Larval IRs did not differ among *p*CO_2_ levels in both experiments (see Experiments #1 and #2), suggesting that larval feeding is independent of parental acclimation. Similar responses were described for *M. edulis* larvae in this pH range, which showed feeding rates that were notably robust to increasing seawater acidity, thus suggesting that the cost of energy maintenance could have been supplied from other physiological processes such as energy storage (Ventura et al., 2016). However, our findings must be validated using a larger sample size or comparative methodological approaches (i.e., Coulter counter, or citometry). Typically, this kind of complex experimental design should be assessed with a larger number of bottle replicates (e.g., >5).

Temperature affected the physiological performance of larval stages depending on parental exposure. From broodstock exposed to high *p*CO_2_ conditions, we obtained larger larvae (shell length) with higher IRs at 15°C, which suggest that increasing temperature can promote an increase in energy intake. Previous studies in marine invertebrates have shown an antagonistic relationship between OA and temperature (Byrne and Przeslawski, 2013). Other studies have observed a positive effect of temperature on the physiology of juvenile *M. chilensis* under future OA scenarios, although using a slightly higher temperature range (12–16°C) than those applied in this study (11–15°C; e.g., Duarte et al., 2014; Navarro et al., 2016). For instance, Duarte et al. (2014) observed an increase in calcification and growth rate a high temperature, whereas Navarro et al. (2016) observed an increase in clearance, absorption efficiency, and scope for growth in juvenile individuals. Duarte et al. (2014) proposed this range (12–16°C) to simulate the potential increase in seawater temperature predicted by the IPCC (2007) for the late 21st century. Nevertheless, these ranges of sea surface temperature fall into the natural variability range measured currently in a typical mussel farming area in Chiloe Island, Southern Chile; therefore, it cannot be considered as a realistic ocean warming study (Narvaez et al. submitted to Progress in Oceanography).

The combined effect of broodstock exposure to high *p*CO_2_ levels and larval rearing under high cadmium concentrations resulted in decreasing larval IRs. Poulsen et al. (1982) found non-significant effects of similarly high cadmium concentrations on the feeding rates and growth of *M. edulis* juvenile. However, in our preliminary experiment, we found a significant drop in IRs for 21-days-old larvae when exposed to high cadmium concentration (10 μg L^-1^). Nevertheless, this cadmium concentration is much higher than Cd concentrations found in mussel farming areas (<1 μg Cd L^-1^, unpublished data by MUSELS Research Center) and/or similar tidal inlets and fjords in southern Chile (0.14 μg Cd L^-1^; Ahumada et al., 2011).

Therefore, larval performance and food intake for larval growth is largely determined by the environmental conditions to which brood stock were exposed during its reproductive stage. Almost all of the parameters evaluated in this study changed as a function of broodstock environment. However, variation in multiple stressor scenarios has diverse effects on the physiological plasticity of the offspring, making it difficult to generalize as to how OA and other environmental stressors affect offspring phenotype in marine invertebrate larvae offspring. Finally, we hope that this kind of experimental approach will stimulate other researchers to examine not only phenotypic plasticity in life history, physiological traits, and tradeoffs, but also intergenerational effects, which are far more informative regarding the selective and evolutionary consequences of parental effects on offspring phenotypes beyond genetic inheritance.

## Ethics Statement

This study was carried out in accordance with the recommendations of the Ethic Committee from the Universidad de Concepción, Chile. The protocol was approved by the institution of authors.

## Author Contributions

RD, CV, and ML designed the experiments, participated in data analysis/interpretation, and performed the writing of the manuscript. RD carried out the experimental studies and data acquisition. FT and ET contributed to intellectual content, revising critically, and manuscript editing. All authors approved the final version of the manuscript.

## Conflict of Interest Statement

The authors declare that the research was conducted in the absence of any commercial or financial relationships that could be construed as a potential conflict of interest.

## Footnotes

1 www.ceazamet.cl

## Supplementary Material

TABLE S1 Data report for experiment 1 and 2; including larval ingestion rates, larval shell length, broodstock metabolism and ingestion, and oocyte diameter.
